# Exploratory palynological studies at the Tell el-Daba'a-Avaris archaeological site

**DOI:** 10.1371/journal.pone.0180770

**Published:** 2018-02-07

**Authors:** Mohamed F. Azzazy

**Affiliations:** Surveys of Natural Resources Department, Environmental Studies and Research Institute University of Sadat City, Sadat City, Egypt; Seoul National University College of Medicine, REPUBLIC OF KOREA

## Abstract

A palynological study of samples collected from the Tell el-Daba'a (Avaris) archaeological site, the capital of the Hyksos located in the Northeastern Nile Delta, Egypt, was conducted. A range of samples were analyzed for pollen content: mudbricks from tomb walls dating from the Middle (cal. 2124–1778 BC) and New Kingdom ages (cal. 1550–1750 BC), kitchen remains dating from the Middle Kingdom, kitchen middens from the 19th Dynasty (cal. 1750–1058 BC), and tomb offering jars from the Late Period (cal.1000-600 BC). Floristic composition of modern vegetation analysis at different habitats revealed four community types and nine associated types, providing an indicator of high soil salinity and moisture content. Cereal and *Achillea-*type pollen were common in the mudbrick samples, indicating the probable use of these plants as temper during mudbrick manufacturing in the Middle and New Kingdoms. The kitchen samples were dominated by cereals, broad bean, celery, and other weed pollen types, indicating the importance of cereals, legumes, and celery as strategic crops for food or medicines during the Middle Kingdom period. Weed pollen types were probably associated with crops, with “*Cheno-am*” pollen type recorded at highest abundance in the tomb filling jar, which may indicate the use of these aromatic herbs to repel insects and animals from tombs.

## Introduction

Tell el-Daba’a (the name for Avaris, the capital of the Hyksos), located in the northeastern Nile Delta, has been recognized as an archaeological site since 1885 and contains accumulations of ancient settlement debris [1]. A number of excavations have revealed information about the gradual settling of Asiatic immigrants in the Delta under the Hyksos, a group of people who ruled for over a century during the Second Intermediate Period of Egypt (c.1650-1550 BC) [2]. The Hyksos forged a strong power base in the Northeast Delta, an area of great strategic importance for control of critical trade routes with the Near East and the Mediterranean by both land and sea. Tell el-Daba’a is also associated with Pi-Ramesses, the Delta residence of Ramesses II in the 19th Dynasty [1].

The archaeology at Tell el-Daba’a provides numerous indications of foreigners throughout the stratigraphy which can be used, in addition to previous interpretations, to establish a relative timeline of occupation and migration. Stratum e/1-3 contains material of a purely Egyptian cultural context dating to the early twelfth dynasty, whereas Stratum H = d/2 exhibits evidence of the first newcomers, after a hiatus, who were already Egyptianised. Syrian ‘Mittelsaal’ houses and a ‘Breitraum’ house give an indication of the origin of the inhabitants along with burials yielding foreign weaponry and donkey burials typical of contemporary Syrian traditions. With finds of distinctive Mbiia Levantine painted ware and jugs of Syrian types, such evidence shows interactions parallel to other late 12^th^ Dynasty sites both in and outside Egypt [3].

Eduard started excavations in the area in 1885. Between 1929 and 1939, Pierre Montet excavated at Tanis, 20 km to the north, finding remarkably rich tombs. He believed that he had found the location of Avaris, and his opinion was widely accepted at the time. Yet others, such as Labib Habachi, one of the pioneering Egyptian Egyptologists, were not convinced. In 1941–42, he excavated at Tell el-Daba’a for the Egyptian Antiquities Service and came to the conclusion that this was in fact Avaris: when a detailed study of the topography of the site and its surroundings was made by Manfred Bietak of the Austrian Archaeological Institute in the 1980s, Habachi's hypothesis was confirmed. Bietak's mission revealed that the actual Hyksos capital was indeed at Tell el-Daba’a [4].

Archaeological palynology examines human uses of plants in the past. This can help determine seasonality of site occupation, presence or absence of agricultural practices or products, and 'plant-related activity areas' within an archaeological context. Bonfire shelter is one such example of this application [5]. Although the Nile Delta is of major archaeological importance, there are relatively a few palynological studies of an area to provide understanding relationship between humans and their landscape [6]. Fossilized pollen grains of cereals type are a useful tool for reconstructing agricultural activities and food use [7]. For example, [8] examined the pollen content of three samples taken from mudbrick at the Giza Pyramid area, but this provided only limited information about the local agricultural conditions and only provided proof-of-concept for extraction of pollen from ancient Egyptian mudbrick. Due to the practically conterminous accumulation of sediments in the Nile Delta, evidence for early settlement as well as for environmental conditions during the past is hidden below the surface. Therefore, palynological studies of deposits are a necessity.

The application of pollen analysis to archaeobotanical questions remains limited. Here we performed an archaeopalynological study of mudbrick and other samples obtained from the Tell el-Daba'a site to investigate the flora, vegetation, agricultural activities, and food use in ancient Egyptians in the Nile Delta. Specifically, the archaeological record is the body of physical (not written) evidence about the past. It is one of the core concepts in archaeology, and the academic discipline concerned with documenting and interpreting the archaeological record [8], while the archaeological record can consist of the earliest ancient findings as well as contemporary artefacts. Human activity has had a large impact on the archaeological record. Destructive human processes, such as agriculture and land development, may damage or destroy potential archaeological sites.

Our objectives were to: (i) investigate present, ancient, and tomb flora; (ii) clarify ancient Egyptian thinking about herbal medicines; and (iii) understand agricultural activities and food use in Ancient Egypt.

## Materials and methods

### The study area

Tell El‐Daba'a is one of the famous and important archeological sites in the eastern part of the Nile Delta, Egypt. It is located at about 7 km north of Faqous city, Sharqiya Governorate. The earliest evidence of occupation in this area dates back to the first intermediate period, when royal estate was found in the region. During the Middle Kingdom, it was a flourishing settlement area known as Rawaty, “mouth of the two roads”. During the turbulent second intermediate period, it was the capital city of Hyksos (Dynasty XV), with both economic and military roles. During the XVIII^th^ Dynasty, the area was known by the name Peru‐nefer). In the XIX^th^ Dynasty, it became a part of Piramesse the northern residence of Ramsses [9].

### Palaeo-topography of Tell el‐ Daba’a archaeological site

**Fig 1:**
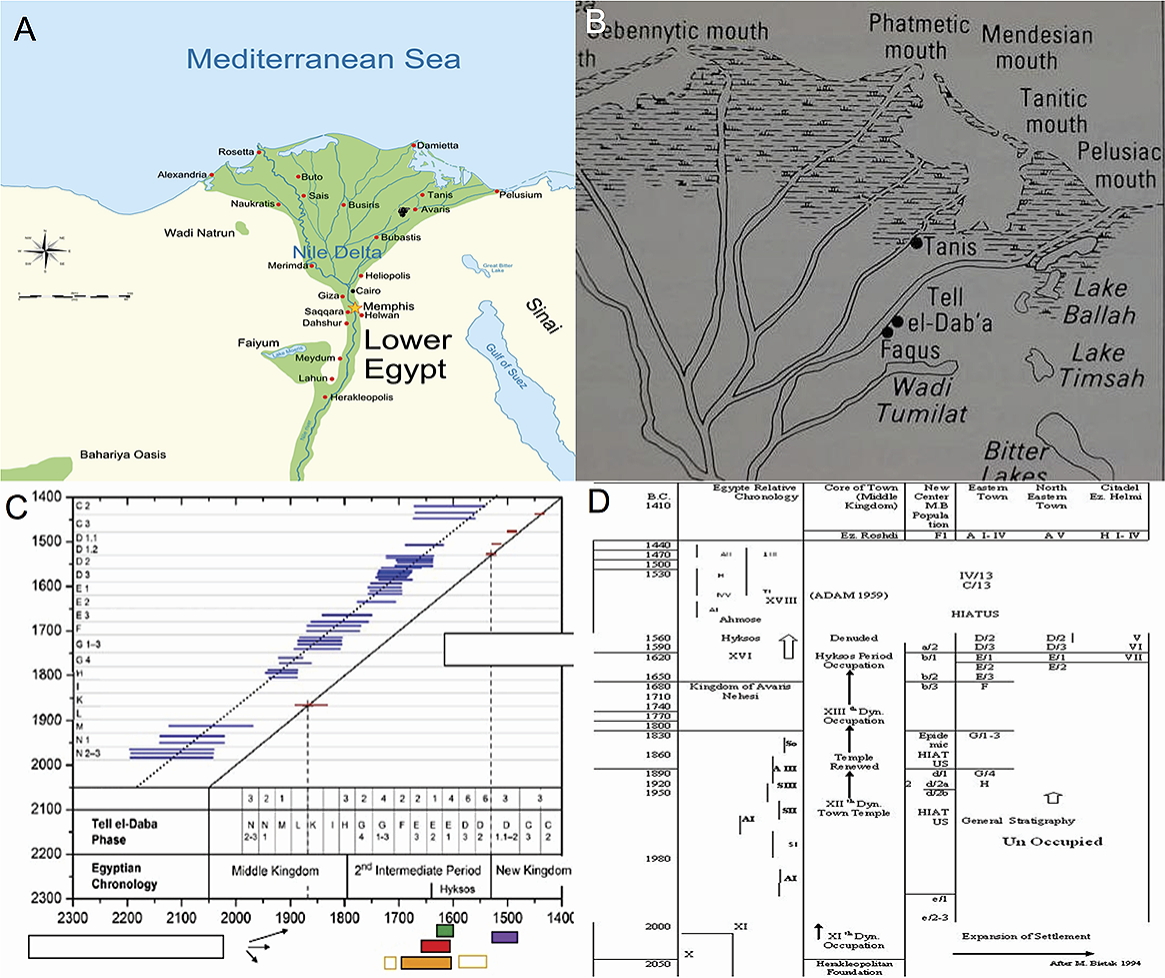
(A) Location of (Tell el-Daba'a) on the Nile Delta. (B) Ancient branches of the Nile. (C) Chronology of Tell el-Daba'a and Egypt. (D) Geology and chronology of the studied strata.

Geophysical and palynological surveys reveal the presence of flood basins, levees, and channel deposits. Low palynomorph concentrations probably result from the high sedimentation rate and mean that further work is needed on the methods for palynological study in the region. The site of Tell El‐ Daba'a, located in the northeastern Nile Delta, has been known since 1885. This part of the Nile Delta is generally characterized by a low alluvial plain with southwest-northeast trending belts of higher ground known as *geziras* (Arabic: sand-islands) and archaeological sites known as tells which are accumulations of ancient settlement debris. Excavations at the site have been conducted since 1966. Tell el‐ Daba’a is connected with the capital Avaris lying below deposits of silt and modern cultivation. Excavations yielded information about the gradual settling of Asian immigrants in the Delta under the so-called Hyksos, foreign rulers who held sway for over a century during the Second Intermediate Period *(c*. 1650–1550 BC) [9]. Tell el-Daba'a is believed to be ancient Avaris, capital of the Hyksos [1], on the eastern margin of the Nile Delta (**Fig 1A**).

The area has seen extensively excavated by an Austrian team led by Bietak [10], where Hyksos war wheels were excavated. Historically, Herodotus mentioned five Nile delta river branches (**Fig 1B)** [11]. The eastern branch is the Pelusiac branch, so called because of the city of Pelusium, which has since dried up with only scarce remains apparent; the Palaeo- Pelusiac branch was located close to Avaris according to archaeological surveys [10,12]. The river lay west of the city between the modern villages of Enbalaissa, Madrabi, and Ezbet Helmi. The main branch appears to separate into two channels in the north around the ancient city of Piramesse, where lithology linked to the presence of palaeo-lakes or river branches can be observed.

The nature of civilization at Tell el-Daba’a is understood from archaeological examination of the site. Excavations have discovered buildings, namely residences, tombs, and temples that combine Egyptian and Canaanite architectural styles. The society of Tell el-Daba’a interacted with individuals from other regions who influenced their frescos. Although the remains have been damaged by the marshy environment as well as by the continual rebuilding and agriculture on the site, archeologists have shown that this city was occupied by a wealthy society with a large sacred precinct and unusual burial practices [13]. From 1951 to 1954, Shehata Adam partly excavated the 12th Dynasty site of Ezbet Rushdi near Tell El-Daba’a (**Fig 1C and 1D**).

Approximately 500 pieces of Cypriot pottery, containing oil and perfume, were discovered at Tell el-Daba’a. Pendent Line, Cross Line, and White Painted V styles of White Painted Cypriot pottery compose the largest component of exported pottery to Tell el-Daba’a, indicating that Tell el-Daba’a had trade relations with Cyprus. A single rim fragment from a jar of the White Painted V Fine Line Style was found at the site [14]. The Hyksos citadel must have been constructed towards the end of the Hyksos Period (ph. D/2).

### Structures and organisation of the city of Avaris

The focal point following initial excavations shiftsed from palaces to real settlement archaeology, meaning that urbanistic considerations were in the foreground. An excavation begun in autumn 2010 in Ezbet Rushdi. The original assumption that this area was occupied by houses of the late Second Intermediate Period with graves had to be surprisingly revised in the course of the campaign in autumn 2010. An extensive development was uncovered covering at least 700 m², divided into groups of rooms with massive walls and courtyards. Grain storage areas and ovens could be identified in the courtyards. The function of this development or city quarter has not yet been finally determined: it probably represents an administrative building (installation/domain) or an economic installation, which might be indicated by the large number of seal impressions found here [15].

### Anthropic features

Two urban areas formed the aim of recent salvage excavations at Tell el-Daba’a—Avaris (Nile Delta, Egypt). Rushdi III, situated in the north-east of the site, was excavated from 2010–2012. The trench, measuring about 60 x 50m, yielded residential quarters of varying sizes, separated by streets. In the western part, larger buildings, possibly with administrative functions, prevail. Here, the settlement started already in the 15th Dynasty and was never overbuilt until its abandonment in the late 2nd Intermediate Period. The excavation yielded a varied set of contexts: room fills, street layers, fireplaces, granaries, small pits, floors, walls and other types of deposits. What was originally believed to be a river harbour turned out to be another settlement area, with some funeral structures, of the 2nd Intermediate Period, which is partly disturbed by Ramessid buildings. There is a clear upper limit of fragment size in the animal remains found. Most contexts favored the preservation of small specimens, containing remains indicative of taphonomic stress (teeth, shaft splinters) and delicate bones of birds and fish. At Rushdi IV, samples are totally dominated by the bones of birds and fish with the domestic compounds (silos, pits) [9].

### Geology and chronology of the studied strata

The chronology and nomenclature used at the Tell el-Daba'a site is shown in (**Table 1**and **Fig 1C** and **1D**). Pleistocene age of this stratum was confirmed by two optically stimulated luminescence (OSL) ages in core AV-02 in layer A: 15200±1600 BP at the base (AV 54–7) of the sequence and 12100±1100 BP at the top (AV 54–5). On top of this Pleistocene sequence, a layer quite similar to layers A and B from a grain-size point of view is present. Layer C of core AV-02 was dated (AV 54–4) to 7910±830 BP (6740–5080 BC), thus Holocene. According to [16], the Pelusiac branch was not active at the time. This explains why the sedimentary facies do not change much between 15000 and 8000 BP: the deposition processes themselves are probably the same. The layer D in drilling AV-02 shows a clear sedimentary change. This sequence is also observed in boreholes AV-03, 02, 04, and 62 and does not appear anywhere else at the Avaris site, especially considering its thickness (up to 4 m). It presents a grain-size distribution and an organic component [17]. This kind of deposit is also typical of well-protected environments, and is often found in harbor sediments [18,19].

**Table 1:** Collected samples specification.

Sample	Type of sample	Archaeology	Lithology (strata)	Chronology
1	Mudbrick	Pelusium	R/1- L/61 str K.	12^th^ Dynasty Sesostris 111 about (1900 BC)
2	Mudbrick	Pelusium	R/1- I/61, str C	Sesostris 111.(2124–1778 BC)
3	Mudbrick	Pelusium	R/1- L/61 str K	Late 12^th^ Dynasty. About (1820 BC)
4	Mudbrick	Pelusium	H/111- Q/18- PL3. Wall D2	18^th^ Dynasty. New Kingdom about (1750 BC)
5	Mudbrick	Pelusium Hyksos- XVI	H/111-r/18- PL3. Wall str 1	18^th^ Dynasty. New kingdom about (1550 BC)
6	Kitchen Remains	Hyksos- XVI	H/III-Q/19.L7 str B.	11^th^ Dynasty. Middle Kingdom. (2124–1778 BC)
7	KitchenRemains	Hyksos- XVI	No2.H/III-Q/19, L7.str.b B	19^th^ Dynasty. New Kingdom (1750–1058 BC)
8	Tomb filling Jar	Hyksos- XVI	A/II, P15.pl3-N/2wa	Late period (1000–600 BC)
9	Tree pit	Hyksos- XVI	R/1-I/61Sur	19^th^ Dynasty. New Kingdom (1750–1085 BC)

### Modern vegetation analysis

Vegetation analysis was carried out on four community types representing the vegetation of the study area (**Table 2**). In each community, 2 stands (10m x 10m) were studied. The vegetation analysis and floristic composition were carried out according to [20]. Within each stand, plant species were recorded.

Taxonomic nomenclature followed [21], updated with [22]. Plant cover was estimated quantitatively, Abundance (ab %) by the line intercept method [23] according to the equations:

$$Abundance:\;ab\%=\frac{No.individuals\;of\;a\;given\;species\times 100}{Total\;No.of\;all\;individuals}$$

was used.

**Table 2:** Present plant vegetation cover surrounding the study area at Tell el-Daba'a (Avaris archaeological site).

Dominant Species	Associates	Number of Individuals	Abundance %
***Imperata cylindrica L***	*Medicago sativa*	7	25.0
	*Gnaphalium luteo-album*.	3	10.7
	*Lamium amplexicaule*	5	17.85
	*Sonchus oleraceus*.	3	10.7
	*Cyperusrotundus*	4	14.28
	*Amaranthus graecizans*	6	21.42
***Chenopodium ambrosides L*.**	*Malva parviflora*	5	9.0
	*Cichorium endivia*	15	27.0
	*Brassica nigra*	20	36.36
	*Avena fatua*	10	18.1
	*Amaranthus ascendens*	5	9.0
***Conyza bonariensis (L*.*)* Cronq**.	*Phragmites australis*	23	46.0
	*Cyperus articulatus*	5	10.0
	*Cyperu srotundus*	12	24.0
	*Anethum graveolens*	10	20.0
***Polypogon monspeliensis*** (***L***.) ***Desf***.	*Cynodon dactylon*	27	53.0
	*Emexs pinosa*	9	17.0
	*Launaea fragilis*	6	21.0
	*Convolvulus arvensis*	5	10.0

### Sample collection

**Fig 2:**
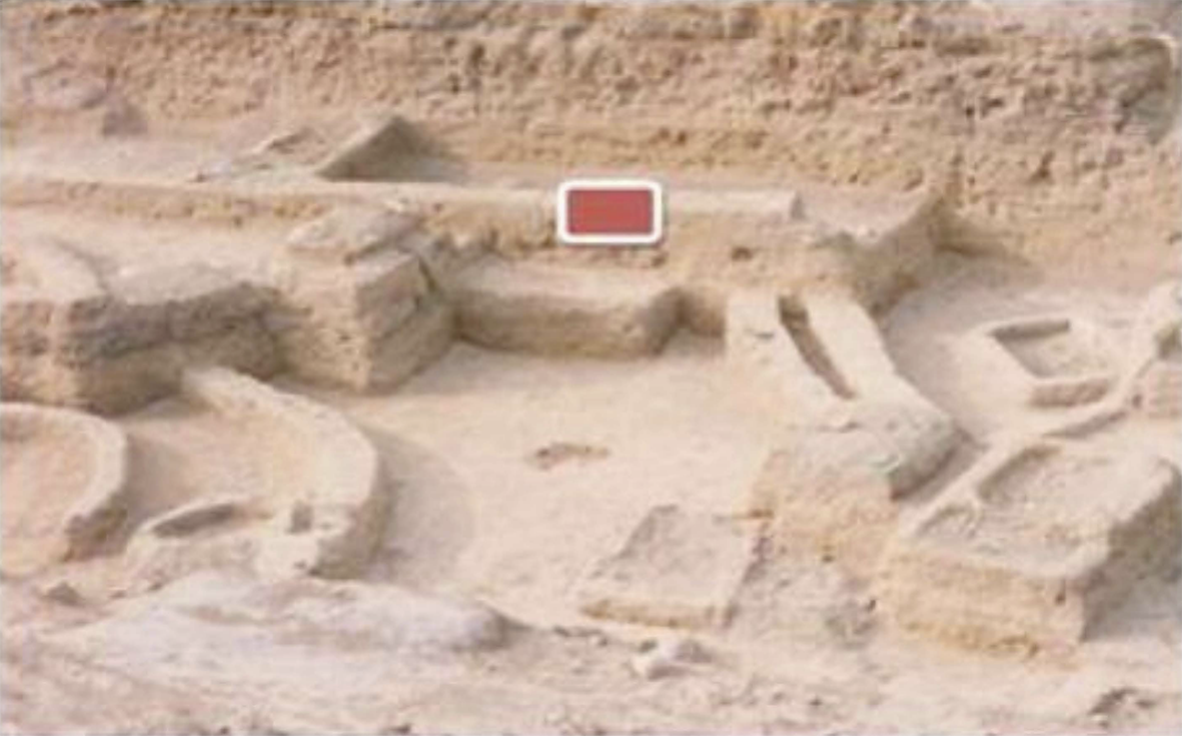
Predynastic house with mudbrick walls.

**Fig 3:**
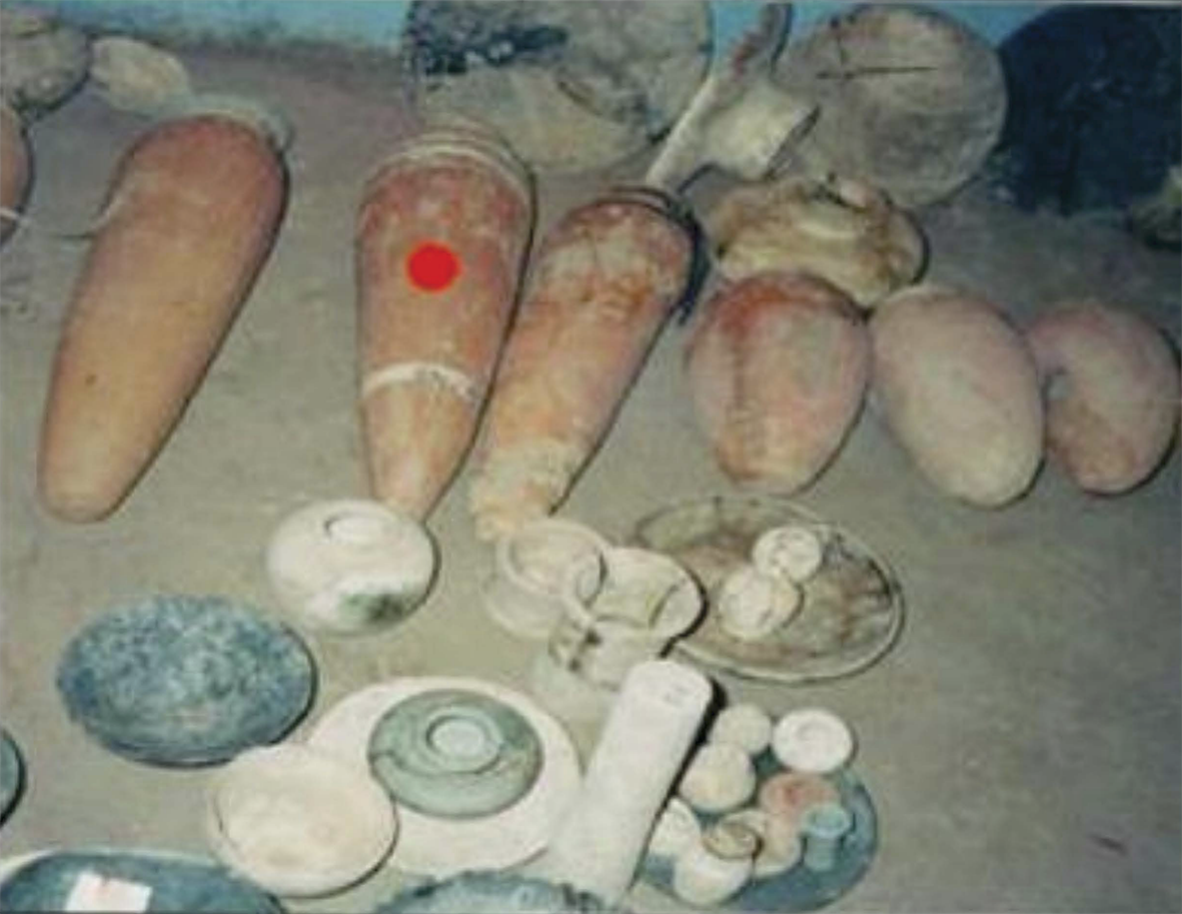
Pottery pots and jars excavated from the Tell el-Daba'a archaeological site.

Samples were collected in spring 2013. The mudbrick samples collected for pollen analysis were taken from an excavated area near to a prominent granite temple beside the Smaller Palace Ezbet Helmi and Tell el-Daba’a (**Fig 2**). The excavation in this area revealed houses with mudbrick walls; these samples had clearly identified chronologically by the archaeological Austrian mission excavations in Egypt and the Austrian Archaeological Institute of Egyptology Professor Manfried Bietak. The outer layer of the enclosed mudbrick, which might have been contaminated with present day pollen by the action of rains and wind, was removed with a knife. Samples were then taken from the interior part the mudbrick with forceps, taking precautions not to be contaminate with recent pollen. Kitchen samples (**Fig 3**) were collected from the pits, residue of houses and some remains, e.g., wood ash, seed containers, ashes from kitchen fires, food remains, and broken jars.

### Sample preparation

Five grams subsamples from each sample were extracted for their fossil pollen using a monofilament sieve according the method of [7]. Three gram subsamples were placed in boiling thermoplastic tubes, mixed with 10 ml KOH (10%) using the Erdtman technique [24,25] and placed in a boiling water bath for 15 minutes. The samples were sieved through a 100 um mesh aperture. The pollen grains were passed through a monofilament sieve and pellet washed with distilled water then centrifuged and decanted. Washings were then made up with distilled water and centrifuged at 3000 rpm for three minutes centrifuged and decanted. The liquid was decanted and 10 ml hydrofluoric HF (40%) added, placed in a boiling water bath overnight, centrifuged, and decanted. The pellet was resuspended in 10% HCl to dissolve residual silicoflorides, centrifuged, and decanted. The pellet was resuspended in glacial acetic acid acetolyzed by standard acetolysis with anacetolysis mixture (9:1 acetic anhydride: concentrated sulfuric acid) to dehydrate after acetolysis. Acetolysis was according to [7]. Samples were mixed with a known number of marker grains (Lycopodium tablets) to determine the concentration of any identified pollen grains [26]. Samples were stained with safranin. Samples were processed for microscopy were stored in Glycerol Jelly (mixture of glyccerine, gelatin and phenol) to avoid microbial decomposition.

### Pollen analysis

**Fig 4:**
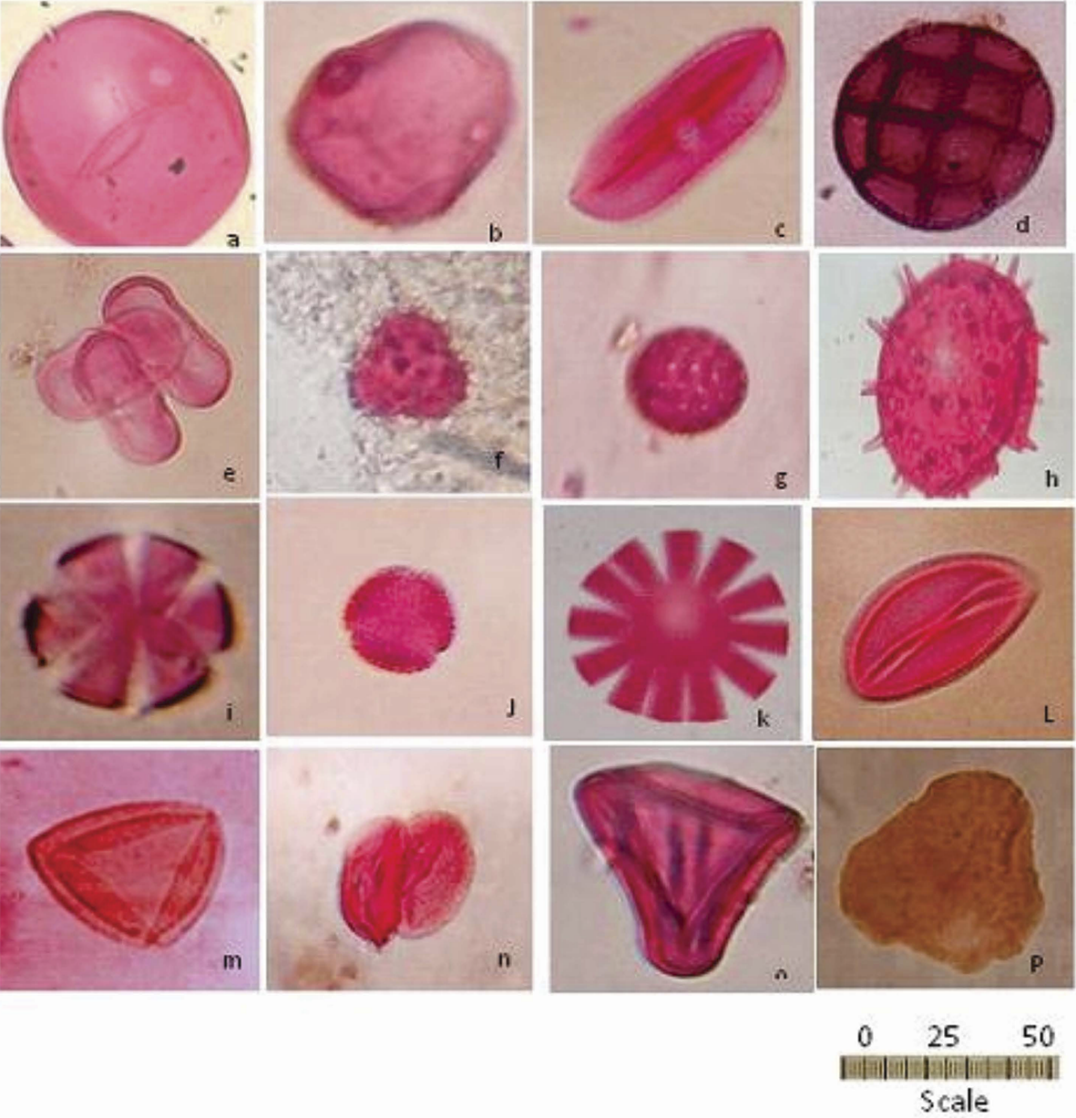
Representative photomicrographs (x 1000) of the pollen spores identified. (a) cereals type 45 um, (b) wild grasses 28 and 43 um, (c) *Viciafaba* 45 um, (d) *Acacia* type 48 um, (e) cluster of *Apiaceae* type 33 um, (f) *Achillea* type 32 um, (g) *“Cheno-am”* type 30 um, (h) *Malvaceae* type 49 um, (i) *Lamiaceae* type 36 um, (j) *Olea* type 24um, (k) *Sesame* type 49 um, (l) date palm 29 um, (m) *Nymphaea* type 48 um, (n) *Typha* type 39 um, (o) *Cyperus* type 43 um, (p) *Lycopodium* spore 50 um.

Pollen analysis was performed using light microscopy at x400 for large pollen and x1000 for small pollen. The number of grains of each pollen taxon were counted and used to produce a pollen diagram, up to 300 grains per sample. Standard textbooks [7,27,28] and a reference collection of most pollen grains were kept in the Environmental Studies and Research Institute (ESRI), Sadat City University, were used for identification. For distinguishing Cereal pollen type from wild grass pollen was performed according to [29] and Andersen [30], which depends on Annulus diameter and size in pollen identified by using the method in [31] of distinguishing cereals from grasses. *Chenopodium* and *Amaranthus* were denoted “*Cheno-am*”. Pollen and spore nomenclature follows [32]. A *Lycopodium* tracer was added to calculate pollen concentration (grains/cm; at least 300 pollen/slide). Pollen grains that were broken, corroded, hidden, or otherwise damaged were counted as 'indeterminate' and those that were unidentifiable were counted as 'unknown’. Photomicrographs of fossil pollen were taken. Difficult to identify pollen were sent to Professor Sekena Ayyad, Botanical Institute of the Bergen University, Norway, for identification. The identified pollen are shown in (**Fig 4**).

## Results

### Present plant vegetation cover surrounding the study area Tell el-Daba'a

The composition of the present plant cover of the study area is shown in (**Table 2)**. Four dominant plant species were recorded: *Imperata cylindrica L*., *Chenopodium ambrosides L*, *Conyza bonariensis (L*.*)* Cronq and *Polypogon monspeliensis* (*L*.) *Desf*. Different pollen were associated with each dominant species for *Imperata cylindrica L*,: *Medicago sativa*, *Gnaphalium luteo- album*, *Lamium amplexicaule*, *Sonchus oleraceus*, *Cyperus rotundus*, and *Amaranthu sgraecizans* at abundances of 25.0, 10.7, 17.85, 10.7, 14.28 and 21.42%, respectively; for *Chenopodium ambrosides L*.:*Malva parviflora*, *Cichorium endivia*, *Brassica nigra*, *Avena fatua*, and *Amaranthus ascendens* with abundances of 9.0, 27.0, 36.36, 18.1, and 9.0%, respectively; for *Conyza bonariensis (L*.*)* Cronq: *Phragmites australis*, *Cyperus articulates*, *Cyperus rotundus*, and *Anethum graveolens* with abundances of 46.0, 10.0, 24.0, and 20.0%, respectively; and for *Polypogon monspeliensis* (*L*.): *Cynodon dactylon*, *Emex spinosa*, *Launaea fragilis*, and *Convolvulus arvensis* with abundances of 53.0, 17.0, 21.0, and 10.0%, respectively.

### Mudbrick samples

**Fig 5:**
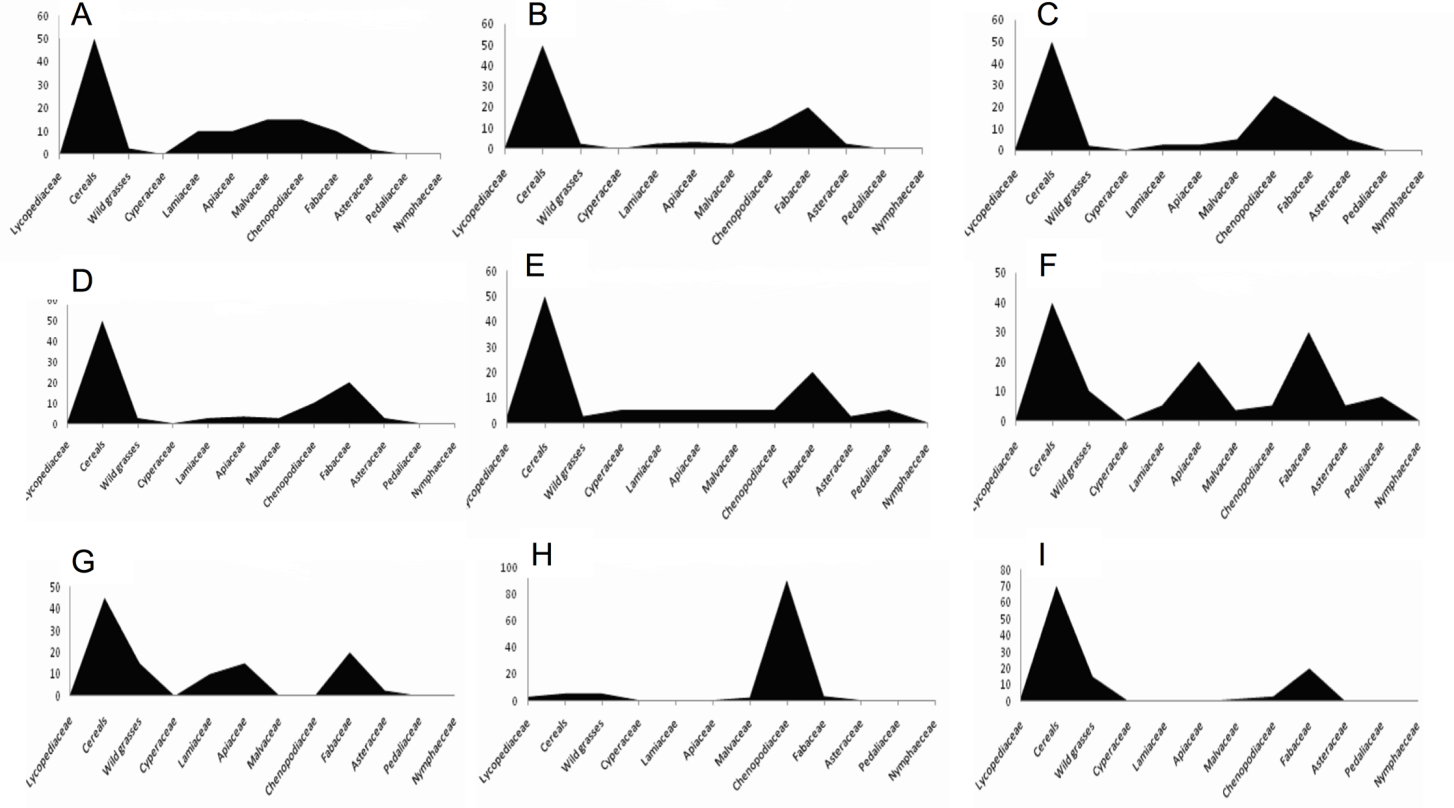
(A) Mudbrick R/1- L/61 str K., 12th Dynasty Sesostris 111 about 1900 B.C. (B) Mudbrick H/111- Q/18- PL3. Wall D2, 18th Dynasty. New Kingdom about 1750 BC. (C) Mudbrick R/1- L/61 str K, Late 12th Dynasty. About 1820 BC. (D) Mudbrick H/111- Q/18- PL3. Wall D2, 18th Dynasty. New Kingdom about 1750 BC. (E) Mud brick H/111-r/18- PL3. Wall str 1, 18th Dynasty. New Kingdom about 1550 BC. (F) Kitchen remains H/III-Q/19.L7 str B., 11th Dynasty. Middle Kingdom. 2124–1778 BC. (G) Kitchen remains No2.H/III-Q/19, L7. strbB, 19th Dynasty. New Kingdom 1750–1058 BC. (H) Tomb filling jar A/II, P15.pl3-N/2wa, Late period 1000–600 BC. (I) Tree pit R/1-I/61Sur 19th Dynasty. New Kingdom/ 1750–1085 BC.

The relative abundances of fossil pollen grains retrieved from five mudbrick samples are shown in (**Table 3**; **Fig 5A–5E**). Cereals type dominated all samples (**Table 3**), with relative abundances of 50, 55, 50, 50, and 45%, respectively. Fabaceae (Leguminosae) *Vicia faba* (broad bean) shown in (**Fig 4C**) pollen was present in all but one sample (15, 20, 25, 20 and 20%) respectively. Asteraceae (Compositae) (**Fig 4F**) pollen was recorded in four samples (10, 10, 15, 2.5 and 0%) respectively, and Apiaceae (Umbelliferae) (Fig 4E) was recorded in 4 samples (10, 2.5, 2.5 and 10%). “*Cheno-am*” pollen type (**Fig 4G**) was recorded in all samples (10, 5, 5, 2.5, and 5%), while Cyperaceae (sedge family) (**Fig 4O**) was also recorded in all samples (2.5, 2, 2, 2.5, and 2.5%) respectively (**Table 3**).

**Table 3:** Relative abundance of different pollen and spore types at Tell el-Daba'a (Avaris archaeological site).

Chronology	Sample type	Gymno	Monocot		Dicot							
		Lyc	PO	CY	Lam	Apia	Malv	Ch	Fab	Ast	ped	Nym
12^th^ Dynasty Sesostris 111 about 1900 BC	Mudbrick R/1—L/61 str K.	0	50.0	2.5	0	10.0	0	10.0	15	10.0	0	0
Sesostris 111 2124–1778 BC	Mud brick: R/1- I/61, str C	0	55.0	2.0	0	0	10.0	5.0	20	10.0	0	2.5
Late 12^th^ Dynasty. About 1820 BC	Mud brick:R/1- L/61 str K	0	50.0	2.0	0	2.5	2.5	5.0	25	15.0	0	0
18^th^ Dynasty. New Kingdom about 1750 BC	Mud brick: H/111-Q/18- PL3. Wall D2	0	50.0	2.5	0	2.5	3.3	2.5	20	2.5	0	0
18^th^ Dynasty. New Kingdom about 1550 BC	Mud brick: H/111-r/18- PL3. Wall str 1	2.0	50.0	2.5	5.0	5.0	5.0	5.0	20.0	0	5.0	0
11^th^ Dynasty. Middle Kingdom. 2124–1778 B.C	Kitchen Remains H/III-Q/19.L7 str B.	0	400	0	0	0	3.4	0	30.0	0	0	0
19^th^ Dynasty. New Kingdom 1750–1058 BC	Kitchen remains No2.H/III-Q/19, L7.str B	0	45.5	0	0	15.0	0	0	20.0	2.5	0	0
Late period 1000-600B.C	Tomb filling Jar A/II, P15.pl3-N/2wa	2.5	5.0	0	0	0	0	90.0	0	0	0	0
19^th^ Dynasty. New Kingdom/ 1750–1085 B.C	Tree pit R/1-I/61Sur	1.5	70.0	-0	0	0	1.5	12.3	20.0	0	0	0

**Abbreviations:** Lyco = Lycopodium; Gymno = Gymnospermae; Monocot = Monocotyledonae; PO = Poaceae (Graminae); CY = Cyperaceae; Ty = Typhaceae; Dicot = Dicotolydonae; Ch = “Cheno—am”; Fab = Fabaceae (Leguminosae); Ast = Asteraceae (Compositae); Api = Apiaceae- Malv = Malvaceae; Ped = Pedaliaceae; Lam = Lamiaceae; Nym = Nympheaceae.

Cyperaceae family pollen was recorded in mudbrick sample (R/1-L/61-str K, 12^th^ Dynasty, Sesostris III, about 1900 BC) with an abundance of 2.5, 2, 2, 2.5 and 2.5%. (**Fig 5B**), Lamiaceae was recorded in only one mud brick sample (H/III-Q/18-pl3, wall D2, 18th Dynasty, New Kingdom, about 1750 BC) with an abundance of 5% (**Table 3**and **Fig 5D**). Malvaceae was recorded in one mudbrick sample (H/III-r/18-pl3-wall str I, early 18th Dynasty, about 1550 BC) with a relative abundance of 10, 2.5, 3. 3 and 5% (**Fig 5E**). Pedaliaceae (sesame type) pollen was recorded in the one sample with an abundance of 5%. Wild grasses were recorded in all samples with abundances of 15, 5, 5, 2.5, and 5%, while Nymphaeceae (water lily) family pollen was recorded in one sample with an abundance of 5%. *Lycopodium* (clubmoss) was recorded only in one mudbrick sample (H/III-r/18-pl3-wall str.I, early 18^th^ Dynasty, about 1550 BC) with an abundance of 2.5% with little representation of *Nymphaea*, *Typha*, date palm, *Olea*, and *Sesame* pollen types (**Fig 5E** and **Fig 4M, 4N, 4L and 4K**).

### Miscellaneous samples

The relative abundance of fossil pollen grains shown in (**Table 3**and **Fig 5F and 5G**) extracted from kitchen remains of H/III-Q/19.L7-str B, 11^th^ Dynasty Middle Kingdom, 2124–1778 BC showed a dominance of Cereal type (40%) followed by 20% Fabaceae (*Vicia faba* type), 18% Apiaceae, 15% *Acacia* (**Fig 4D**), and 3% Asteraceae pollen. Fossil pollen grains extracted from kitchen middens remains (No2. H/III-Q/19, L7-str B, 19th Dynasty New Kingdom, 1750–1085 BC) (**Table 3**) showed a dominance of cereal type types (45%) followed by 20% Fabaceae (*Vicia type*), 10% *Acacia* pollen type, 15% Apiaceae, 5% Asteraceae (*Achillea* pollen type), and 5% wild grasses. The tomb filling jar A/II.P15.PL3/N/2wa, Late Period 1000–600 BC (**Table 3**, **Fig 5H**) showed 90% “Cheno-am”, 7% grasses, and 3% *Lycopodium* spores. Analysis of the tree pit of R/1-I/61, Late Period 1000–600 BC recorded 70% Cereal type, 20% *Acacia*, 5% grasses, 2.5% Malvaceae type, and 2.5% *Lycopodium* spores (**Fig 5I)**.

## Discussion

Palynology can contribute to information about how people exploited plants in ancient times [33,34]. For example, pollen collected from middens often indicates the types of plants collected and utilized for food or other economic purposes by prehistoric cultures. Fossil pollen found in floor sediment can be used to suggest how rooms were utilized, while scrapings from the inside surfaces of ceramic vessels may include fossil pollen from plants that were stored in or eaten from those vessels. Here we investigated the different types of pollen grains retrieved from mudbrick and samples obtained from living spaces and tombs from the Tell el-Daba'a archaeological site to investigate ancient Egyptian plants, vegetation, habitat, and living activities.

### Present plant vegetation cover surrounding the study area

The study of floristic composition of modern vegetation in different habitats provides an indicator of soil salinity, moisture content and soil reaction [35]. **Table 2**shows the floristic composition of the study area, with its four community types and nineteen associated species.

The first community is dominated by xerophytes e.g., *Imperata cylindrica L*, with the associated *Medicago sativa*, *Gnaphalium luteo-album*, *Lamium amplexicaule*, *Sonchus oleraceus*, *Cyperus rotundus*, and *Amaranthus graecizans*. Xerophytes vegetation is the most important characteristic type in Egypt, and it constitutes a major part of plant life in the Egyptian deserts. Several studieshave described the ecology of the vegetation types and their relationships with soil and climate [36]. There are similar studies on the relationship between desert vegetation and environmental factors [37]. Halophytic vegetation is the second in importance, occupying the inland salt marshes and littoral of the country. There was a halophytic community dominated with *Chenopodium ambrosides L*. with the associated *Malva parviflora*, *Cichorium endivia*, *Brassica nigra*, *Avena fatua*, and *Amaranthus ascendens*.[36]. The reed swamp (halophytic) vegetation community type was dominated by *Conyza bonariensis (L*.*)* Cronq, with the associated *Phragmites australis*, *Cyperus articulates*, *Cyperus rotundus*, and *Anethum graveolens* in the study area. Wherever there was neglected shallow water (saline, brackish or fresh), reeds predominated (**Table 2**). [38] stated that the most widely spread reeds are *Phragmites australis*, *Typha domingensis*, and other common species of the reed swamp vegetation include: *Cyperus articulatus*, *C*. *Rotundus* and *C*. *difformis*. On the other hand Asteraceae, *Cheno-am*, and Cyperaceae are extremely arid vegetation [39], which may suggest extremely arid vegetation at the study area.

One of the aims of this study was to establish whether the pollen detected were derived from ancient vegetation or represented artefact from current plant species. There were few similarities between present and ancient vegetation cover at the study site, with generally mesophytic and dry habitat plants recorded in the present vegetation. In contrast, the pollen and spores retrieved from ancient samples revealed that the climate was likely to be humid and swampy during ancient times (e.g., from the presence of *Lycopodium*, *Cyperus*, and *“Cheno-am”*). This is in agreement with [40], who stated that members of Cyperaceae, Asteraceae, *“Cheno-am”*, and Apiaceae species grow in wet or dry places while *Lycopodium* grows well in a variety of wet and dry climates [41]. Taphonomy can aid in the understanding of past environments [42], and when studying the past it is important to gain contextual information in order to have a solid understanding of the data. On the other hand, pollen grains that were broken, corroded, hidden, or otherwise damaged were counted as 'indeterminate’ and this damage may due to the high soil pH values are the most destructive factors for pollen preservation conditions [43]. Taphonomic processes allow researchers of multiple fields to identify the past of natural and cultural objects.

### Archaeological pollen (archaeo-palynology)

#### Mudbrick samples

Cereals pollen type and broad bean were abundant in mudbrick samples, perhaps indicating that the chopped straw of these plants was used domestically in mudbrick manufacturing and reflecting the cultivation of cereal crops during these periods. Chopped Fabaceae straw is likely to have been added to cereals as a temper, similar to Ayyad’s findings in the Saqqara tombs. [44] found a similarly diverse range of pollen grains in mudbrick samples from the Pyramid area (2545–2457 BC). Mudbrick analysis can provide valuable information, but caution is required in interpretation. For example, the raw materials may differ and may consist of mud mixed with manure or different pollen types may be included from water sources. It is always better to coordinate pollen data obtained from adobe with other data [45].

Apiaceae (Celery family) pollen was retrieved from three samples (10% abundance) dating to the New Kingdom period (about 1750 BC) and 1550 BC, the presence of this pollen type with cluster forms perhaps indicating that Apiaceae plants were widely cultivated at this time. Asteraceae fossil pollen was greatest in samples marking the late 12th Dynasty (about 1820 BC), and [46] reported that Asteraceae pollen types are indicative of a warm climate. “*Cheno-am*” (goosefoot) pollen was recorded in all samples, with the highest abundance during Sesostris III (about 1900 BC). “*Cheno-am*” pollen may indicate the presence of saline habitats with salt-loving plants in the sediment [47,48]. Cyperaceae was recorded in one sample during Sesostris III (about 1900 BC), perhaps indicating the presence of reed swamps during this period [49], although Lamiaceae was also recorded in one sample during the New Kingdom period, indicating a more moderate mesophytic condition. In this regard, palynology is an extremely useful tool for investigating fluctuations in vegetation that might be associated with climate change and/or human impact [50]. Tell el-Daba'a (Avaris) area was occupied from the early Middle Kingdom to the Roman Period with a hiatus during the 3rd Intermediate Period. Avaris was one of the largest cities in the Nile Delta, and a city of such importance had to be accessible by ship from the rest of the kingdom. Indeed, archaeological findings, such as Minoan-inspired frescoes, reveal that the city was in a relationship with the entire Mediterranean world [51]. This communication meant that the city had a sizable harbor. Furthermore, the Kamose stele mentions that Avaris was used to moor the military fleet of the Hyksos [52]. Until recently, the precise location of the Avaris harbor basin was unclear. The first observed sequence in the cross-section suggests a Late Pleistocene chronology because of its well-known lithology, a vertical alternation of sandy and compact grey silty layers, presenting gypsum concretion. This is similar to the observations made in the North of the Delta by other authors [52].

Malvaceae (mallow family) pollen was recorded in a sample from the early 18th. Dynasty (about 1550 BC), maybe indicating the growth of weeds during this period. Pedaliaceae (sesame type) was recorded in one sample from the 18th Dynasty, New Kingdom (about 1750 BC). Indeed, [51]’s excavations reported that olive and sesame were the primary dietary fats and oils during this period.

Wild grasses were recorded in all samples, with the highest value of 15% observed in a sample from Sesostris III about 1900 BC identified by [32]’s method of distinguishing cereals from grasses and indicating that the grassland vegetation was dense and that grazing may have been absent during this period [31]. Nymphaeaceae (water lily family) was recorded in one sample from the Late 12th Dynasty (about 1820 BC), the presence of which perhaps indicating the proximity of Nile tributaries and an aquatic habitat [53]. The *Lycopodium* type recorded only in one sample (early 18^th^ Dynasty, about 1550 BC) may indicate that the climate and habitat were humid during this period [53]. It was interesting to find *Nymphea* pollen type (sacred lotus) in mudbrick samples from Sesostris III, 2124–1778 BC, which may reflect the presence of this plant and known as Nile water, so indicating Nile water and was explained by addition of Nile water to the clay and straw for manufacturing of mudbrick during this period.

Pollen from Nympheaceae is one of the important ones in the records. The species from this family is more close Egyptian culture, and have been used to indicate fertility and love, and some other human expression in Egyptian culture [54–56]. While, the Egyptian idea of sexuality was identified with creation. Being a flower of creation, the flower *Nymphaea* type (water Lily) became linked to human fertility and sexuality. The images of women holding the flower may be hinting at her ability to bear children or that she was sexually desirable, and images of men holding the flower may hint at his potency. It could also be a way to ensure that the person painted would be fertile—and sexy—in the afterlife [56].

#### Miscellaneous samples

Our data clearly showed that cereal types were the dominant pollen type in four samples taken from the kitchen remains from the 11th. Dynasty Middle Kingdom (about 1778 BC), kitchen midden remains (19th. Dynasty New Kingdom, 1570–1085 BC), filling jar (Late Period 1000-600BC), and tree pit (Dynasty New Kingdom, 1570–1085 BC). Cereal type and grasses were distinguished according to Andersen classification [45]. Annulus diameter was considered more reliable for identification of fossil pollen than pollen size because annulus is less modifiable and can be measured on all grains (large and pointed in cereals but small and rough in grasses). Surface sculpturing is also useful for identification, and pollen size is useful in cases where preservation is good or moderately good. Pollen of *Glyceria sp*., *Hordeum vulgare* or *Agropyron repens*, Secale cereals, and *Avena sativa* were identified in Holocene deposits [57].This high quantity of cereal type suggests that barley and wheat were commonly used in different foods during the Middle, New, and Late Periods, and [31] suggested that the ancient Egyptians had a predominantly cereal-based diet in which barley and wheat were commonly prepared as bread, while [58] reported from archaeobotanical data that *Hordeum* (barley) was commonly present was the dominant crop in samples obtained from 2750–2500 BC. *Vicia faba* (broad bean) pollen was also recorded at high abundance (30, 20, and 30% of samples in kitchen remains, kitchen middens remains, respectively) dating to the Middle, New Kingdom, and Late Periods (1000–600 BC). The Ancient Egyptians may have, therefore, used broad beans in their food, in agreement with [59], who reported *Vicia faba* pollen type in Old Kingdom remains at relatively high frequency. The broad bean appears to have been introduced into ancient Egyptian agriculture during the New Kingdom period, but fossil pollen from this crop has also been found at high concentrations at the Mendes archaeological site (the capital of Lower Egypt, 3000 BC). Apiaceae pollen recorded high abundance in samples from kitchen middens (dating to the 19^th^ Dynasty New Kingdom, 1570–1085 BC) Therefore, members of Apiaceae plants such as *Apium graveolens* (celery), *Anethum graveolens* (dill), *Carum carvi* (caraway), *Coriandrum sativum* (coriander), *Foeniculum vulgare* (fennel), *Cuminium cyminium* (cumin), *Pimpinella anisum* (anise), *Ammi visnaga*, and *A*. *majus* are likely to have been used as spices, in hot drinks, or as food additives and medical remedies [54]. Also, reported excavations with evidence of dietary use of both wild and domesticated fruits and nuts including anise, coriander, grapes, olives, and pomegranates. While, the presence of Apiaceae pollen type in clusters form confirmed that the anthers of the same flower may present and massive cultivation of this plant family.

Asteraceae family pollen were recorded in three samples from kitchen remains dating to the 11^th^ Dynasty, Middle Kingdom (about 1778 BC), kitchen middens remains dating to the 19th Dynasty New Kingdom (1570–1085 BC), and from filling jar dating to the Late Period (1000–600 BC) presence of *Achillea* pollen type. 5% abundance in samples from the kitchen midden remains dating from the New Kingdom may indicate the use of *Achillea* as a medicinal herb during this period; indeed, this herb is today used for intestinal and liver disorders. “*Cheno-am*” pollen was recorded at highest abundance (90%) in samples from the tomb filling jars (Late Period 1000–600 BC). This pollen type has been recorded during the Middle Kingdom, recorded from Predynastic to Graeco-Roman times [60]. They may also represent halophytic communities as reported from the late quaternary, late Palaeolithic and Neolithic, early Dynastic ([61] and [62]) of the Nile Delta and compiled in the codex of ancient Egyptian plant remains, with the identified species *Chenopodium ambrosioides* (wormseed) at this high abundance perhaps indicating use of jars containing this aromatic herb inside tombs to repel insects, worms, and harmful reptiles. [63] reported that Ruta and *Chenopodium ambrosioides* were used as anthelminthics, while recently, [64] reported that *Chenopodium ambrosioide s*was used as a febrifuge and anthelminthic. Malvaceae pollen was recorded at low abundance (2.5%) in the sample from the tree pit (Dynasty New Kingdom, 1570–1085 BC). This low abundance may indicate Malvaceae weeds growing as associates in cultivated crops. Wild grasses were also represented in the samples at low abundances, and this may due to agricultural development at this period also may due to the intensive grazing.

## Conclusions

Palynology is a good tool to shed new light on the complex relationships between ancient societies and the land they exploited, but useful and complementary information is also provided by the study of plant microfossil analyses. Mud brick acts as a capsule for the history of agriculture, human impact, plant vegetation cover, and environmental information. Here we show that chopped straw derived from cereals and legumes were likely to be used as the main temper in mudbrick. The presence of Apiaceae pollen in the remains of kitchen middens suggests that celery, dill, caraway, coriander, fennel, cumin, and anise may have been used as spices, hot drinks, food additives, and medicines. *Chenopodium ambrosioides* (wormseed) in tomb filling jars may indicate that tombs contained this aromatic herb to repel insects, worms, and harmful reptiles.
